# Different materials of cranioplasty for patients undergoing decompressive craniectomy: A protocol for systematic review and network meta-analysis

**DOI:** 10.1097/MD.0000000000027936

**Published:** 2021-11-24

**Authors:** Wanchun Yang, Junhong Li, Tengfei Li, Mingrong Zuo, Yufan Xiang, Xingwang Zhou, Jun Zheng, Hao Li

**Affiliations:** Department of Neurosurgery, West China Hospital of Sichuan University, Chengdu, Sichuan Province, PR China.

**Keywords:** cranial implant, cranioplasty, network meta-analysis, systematic review

## Abstract

**Background:**

Cranioplasty is widely applied on patients who has undergone decompress craniectomy (DC) due to intractable increased intracranial pressure and the cranioplasty materials have been on the bleeding edge of biomolecular and material science. This systematic review and network meta-analysis (NMA) will be conducted to comprehensively evaluate the safety and efficacy of different cranial implants for patients with cranial defects due to various reasons.

**Methods and analysis:**

This protocol has been reported following the Preferred Reporting Items for Systematic Review and Meta-Analysis Protocols. The following electronic databases will be searched from the date of database establishment to September 1, 2020: PubMed, Embase, Cochrane Library, Web of Science, China National Knowledge Infrastructure, VIP, and Wanfang. Randomized controlled trials and non-randomized prospective studies focus on cranial implants will be included. Quality assessment will be conducted using Cochrane Collaboration's tool or risk of bias in nonrandomized studies of interventions based on their study designs. The primary outcome will be postoperative early mortality and implant failure while various complications for secondary outcomes. Pairwise and network meta-analysis will be conducted using STATA V.14 (StataCorp, College Station, Texas, USA). Subgroup analyses and sensitivity analyses will be conducted to assess the robustness of the results.

**Ethics and dissemination:**

This systematic review does not require an ethics approval or the need to obtain informed consent. The results will be published in a peer-reviewed scientific journal.

**Protocol registration number:**

INPLASY 202110001.

## 1 Introduction

Cranioplasty, which is defined as the surgical repair of defects in the cranium, is widely applied on patients who has undergone decompress craniectomy (DC) due to intractable increased intracranial pressure.^[1,2]^ The primary goals of cranioplasty should be to rebuild the structure and function of missing bone and provide support to the soft tissues.^[3]^ Cosmetic demand is also a reason to reckon for patients who have cared about their appearance after DC.

The cranioplasty materials have been on the bleeding edge of biomolecular and material science.^[3]^ Autologous bones, including calvarium, rib and tibia, were widely used in cranioplasty. With the advancement of technique in autologous bone preservation, it has been more and more popular among patients with cranial defects. On account of high rate of resorption of autologous or allograft bone, which usually results in structural breakdown and reoperation, alternatives like plastic, metal, and high polymer materials have entered the stage of history.^[2,4]^ Titanium mesh, hydroxyapatite, alumina ceramics, methylmethacrylate, and Polyetheretherketone are common representative alternatives, which are compatible, safe and stable.^[5–7]^ It is speculated that the future directions for cranial implant include molecular biology to aid bone craft healing, combination of autograft bone and alloplast, and development of brand new materials. Post-DC cranioplasty is reported to be associated with complication rates up to 40%.^[8–10]^ Common complications include implants resorption, infection, hydrocephalus, extra-axial fluid collection, seizure, and intracranial hematoma.^[11,12]^ Cognitive function impairment is also reported in some researches.^[13,14]^ Severe complications may result in poor prognosis, even disability and death.

Network meta-analysis (NMA) is a useful and effective tool for evaluating multiple intervention. In NMA, indirect and mixed estimates can be derived through many potential routes, which can enrich the comparisons and make it comprehensively.^[15]^ A systematic review and NMA willd be performed in this assay to comprehensively evaluate the safety and efficacy of different kinds of repairing materials for patients with cranial defects due to various reasons.

## 2 Methods

A systematic review and network meta-analysis will be conducted with principles and methods of Cochrane Handbook.^[16]^ This protocol has been reported in accordance with the Preferred Reporting Items for Systematic review and Meta-Analysis Protocols guidelines (Supplement Digital Content S1).^[17]^ This protocol has been registered with the International Platform of Registered Systematic Review and Meta-analysis Protocols (INPLASY), with the registration number of INPLASY 202110001.

### 2.1 Eligibility criteria

#### 2.1.1 Types of studies

This study will include both randomized control trials (RCTs) and non-randomized prospective studies, which should be available in full papers in peer-reviewed journals. Retrospective studies, case reports, case series or reviews will not be eligible. No language restrictions will be applied.

#### 2.1.2 Types of participants

The current study will include adult patients undergoing cranioplasty after depression craniectomy. Patients included in this study have undergone cranioplasty because of refractory intracranial hypertension resulting of traumatic brain injury, cerebrovascular diseases, and space occupying lesions.

#### 2.1.3 Types of interventions

We will include studies assessing the efficacy and safety of 2 or more of the following material for cranioplasty. The interventions include:

1. autologous bone,
2. allografts,
3. titanium mesh,
4. hydroxyapatite,
5. methylmethacrylate,
6. alumina ceramics,
7. polyetheretherketone, and
8. combination of synthetic and biological grafts.

#### 2.1.4 Outcome measures

The primary outcomes are early mortality and implant failure, mainly resulting from implant rejection and early severe infection. In view of the short interval between operation and adverse events, there is no time restrictions applied on implant failure.

Secondary outcomes will include presence of postoperative infection, implant resorption, intracranial hemorrhage, extra-axial fluid collection, hydrocephalus, neurological dysfunction, and seizures. Reoperation, cosmetic evaluation, and patients’ satisfaction will also be included and evaluated by both subjective and objective tests.

The time point for outcomes will be the longest follow-up time in each study.

### 2.2 Search strategy and study selection

We will comprehensively search objective studies in the following electronic databases: PubMed, Embase, Cochrane Library, Web of Science, China National Knowledge Infrastructure, VIP, and Wanfang. We will also screen ClinicalTrials.gov to include relevant trials in progress. It will also be necessary to manually search reference lists from relevant articles. Search terms are listed in Supplement Digital Content S2.

Firstly, titles and abstracts of all potentially eligible studies will be screened by 2 independent reviewers (FWT and ZSX). Then full-text papers of the remaining studies will be obtained and screened by the same 2 reviewers independently. Only studies meeting the eligibility criteria will be finally included. If studies have duplicate data, only the study with more recent publication date, larger sample size and longer follow-up time will be chosen. Any disagreement between 2 reviewers will be solved by a third reviewer (LTF). The process of study selection will be shown in a Preferred Reporting Items for Systematic review and Meta-Analysis flow diagram.^[18]^

### 2.3 Data extraction

Another 2 independent reviewers (ZMR and XYF) will be assigned to extract data from the included studies, whose consistency and accuracy will be examined by a third reviewer. The following data will be extracted: the first author, study design, year of publication, sample size, gender, age, region, study period, clinical characteristics, types of interventions, outcome measures, quality rating, and risk of bias assessment. If some data cannot be obtained from the articles directly, we will attempt to contact the authors by corresponding e-mail for those data. Any discrepancy will be resolved by a fourth reviewer (ZXW).

### 2.4 Risk of bias assessment

Two independent authors (LJH and ZJ) will assess risk of bias for every single study in accordance with the Cochrane Collaboration tool for RCTs.^[19]^ Seven specific domains will be estimated, which are sequence generation (selection bias), allocation concealment (selection bias), blinding of participants and personnel (performance bias and detection bias), incomplete outcome data (attrition bias), selective reporting (reporting bias), and other bias of all included RCTs.

As regard to non-randomized trials, risk of bias in nonrandomized studies of interventions will be applied by 2 independent reviewers (ZJ and LH), which also contains 7 domains including bias due to confounding, bias in selection of participants into the study, bias in classification of interventions, bias due to deviations from intended interventions, bias due to missing data, bias in measurement of outcomes, and bias in selection of the reported result.^[20]^

### 2.5 Data synthesis and statistical methods

An overview of all selected studies will be narratively displayed, which mainly include interventions and outcomes. Once data processing is finished, both classic pairwise meta-analyses and network meta-analyses will be conducted using STATA V.14.0 software (Stata Corporation, CollegeStation, Texas).

#### 2.5.1 Pairwise meta-analyses and network meta-analyses

Firstly, standard pairwise meta-analysis of all direct comparisons for the considered outcomes will be conducted using random effects model when data are available. Dichotomous data will be expressed as the odds ratio (OR) with 95% confidence intervals based on study-level and pooled results. And standardized mean differences will be performed for functional outcomes where different scales are used. The statistical heterogeneity will be assessed by Cochrane Q statistic and the Higgins I^2^ statistic.^[21]^

Then, NMA will be used to compare all interventions with the same statistic tool. The appropriateness of this analysis is based on the assumption of transitivity and exchangeability of included studies. A Bayesian approach will be applied in all NMA based on modeling guidance from the National Institute for Health and Care Excellence guidelines.^[22–24]^ Results from the NMA will be presented as summary relative effect sizes (hazard ratios or relative risks) and relative 95% confidence intervals for each possible pair of interventions.

#### 2.5.2 Measures for transitivity assumption

Transitivity is the fundamental assumption of indirect comparisons and network meta-analysis, which is based on the theory that studies are sufficiently similar in important clinical and methodological characteristics.^[15]^ As recommended, the plausibility of the transitivity assumption will be evaluated based on the design characteristics and the methodology of the studies included in the network meta-analysis.^[15]^

#### 2.5.3 Measures for inconsistency

Evidence indicates that network inconsistency can best be identified by node-split modeling.^[25,26]^ Both global and local methods will be used to assess the inconsistency between direct and indirect comparison. The design-by-treatment interaction model will be performed to evaluate the global consistency assumption.^[27]^ Each closed loop in the network will also be evaluated using the same method to examine local inconsistency. Then an inconsistency factor will be calculated to estimate the presence or absence of a statistically significant inconsistency (Bayesian *P* < .05).^[28]^

#### 2.5.4 Measures for publication bias

The publication bias will be assessed using Begg's funnel plot and the Egger test.^[29,30]^ Trim and fill analyses will be conducted when the funnel plot is asymmetrical or *P* value of Egger test < 0.01.

#### 2.5.5 Subgroup analyses and sensitivity analyses

Subgroup analysis and meta-regression analysis will be performed in consideration of potential evident heterogeneity or inconsistency. Subgroup analyses are conducted based on the following factors:

1. age at operation,
2. gender,
3. race,
4. region,
5. size of cranial defects,
6. location of cranial defects,
7. primary disease before DC,
8. interval to cranioplasty after DC.

### 2.6 Quality of evidence

The quality of the evidence will be evaluated with the Grading of Recommendations Assessment, Development, and Evaluation framework.^[31]^ This approach contains 4 major steps including presenting direct and indirect treatment estimates for each comparison of the evidence network, rating the quality of each direct and indirect effect estimate, presenting the NMA estimate for each comparison of the evidence network, and rating the quality of each NMA effect estimate, which can provide ratings for the confidence in the estimates of effect for a specific comparison for all outcomes of importance to patients.

### 2.7 Ethics and dissemination of work

This systematic review does not require an ethics approval or the need to obtain informed consent. We are planning to publish this systematic review and network meta-analysis in a peer-reviewed scientific journal and disseminate it widely through the Internet.

## 3 Discussion

To our knowledge, this will be the first NMA that comprehensively compares the safety and efficacy of different materials for cranial repairing in patients undergoing decompression craniectomy. The result of this systematic review and NMA will provide a comprehensive and objective assessment of cranial implants for post-DC patients, hence providing useful and convincing information and evidence to make better decisions. Therefore, this systematic review will be beneficial for wide audience including patients, neurosurgeons, insurers, policy makers, and researchers working in the field of cranioplasty.

## Author contributions

**Conceptualization:** Wanchun Yang, Junhong Li, Hao Li.

**Data curation:** Junhong Li, Tengfei Li, Mingrong Zuo, Yufan Xiang, Xingwang Zhou.

**Feasibility testing:** Junhong Li, Wanchun Yang

**Funding acquisition:** Jun Zheng.

**Methodology:** Yufan Xiang.

**Software:** Xingwang Zhou.

**Validation:** Hao Li.

**Writing – original draft:** Wanchun Yang, Junhong Li, Tengfei Li, Mingrong Zuo, Yufan Xiang, Xingwang Zhou, Jun Zheng, Hao Li.

**Writing – review & editing:** Hao Li.

## Supplementary Material

Supplemental Digital Content
