# Supplementary material for: Different materials of cranioplasty for patients undergoing decompressive craniectomy: A protocol for systematic review and network meta-analysis
Source: Medicine (Baltimore). 2021 Nov 24;100(47):e27936. doi: 10.1097/MD.0000000000027936 (PMC8615338; doi:10.1097/MD.0000000000027936)
Supplement: Supplemental Digital Content [file medi-100-e27936-s001.doc]

**Supplement 1.** PRISMA-P (Preferred Reporting Items for Systematic review and Meta-Analysis Protocols) 2015 checklist: recommended items to address in a systematic review protocol*

| **Section and topic** | **Item** | **Checklist item** | **Check** |
| --- | --- | --- | --- |
|  | **No** |  | **results** |
|  | | |  |
| **ADMINISTRATIVE INFORMATION** | | |  |
|  |  |  |  |
| Title: |  |  |  |
| Identification | 1a | Identify the report as a protocol of a systematic review | Page 1 |
| Update | 1b | If the protocol is for an update of a previous systematic review, identify as such | NA |
|  |  |  |  |
| Registration | 2 | If registered, provide the name of the registry (such as PROSPERO) and registration number | Page 3 |
|  |  |  |  |
| Authors: |  |  |  |
| Contact | 3a | Provide name, institutional affiliation, e-mail address of all protocol authors; provide physical mailing address of corresponding | Page 1 |
|  |  | author |  |
| Contributions | 3b | Describe contributions of protocol authors and identify the guarantor of the review | Page 6 |
|  |  |  |  |
| Amendments | 4 | If the protocol represents an amendment of a previously completed or published protocol, identify as such and list changes; | NA |
|  |  | otherwise, state plan for documenting important protocol amendments |  |
|  |  |  |  |
| Support: |  |  |  |
| Sources | 5a | Indicate sources of financial or other support for the review | Page 6 |
| Sponsor | 5b | Provide name for the review funder and/or sponsor | Page 6 |
| Role of sponsor | 5c | Describe roles of funder(s), sponsor(s), and/or institution(s), if any, in developing the protocol | Page 6 |
| or funder |  |  |  |
|  |  |  |  |
| **INTRODUCTION** |  |  |  |
|  |  |  |  |
| Rationale | 6 | Describe the rationale for the review in the context of what is already known | Page 2 |
|  |  |  |  |
| Objectives | 7 | Provide an explicit statement of the question(s) the review will address with reference to participants, interventions, comparators, | Page 2 |
|  |  | and outcomes (PICO) |  |
|  |  |  |  |
| **METHODS** |  |  |  |
|  |  |  |  |
| Eligibility criteria | 8 | Specify the study characteristics (such as PICO, study design, setting, time frame) and report characteristics (such as years | Page 3 |
|  |  | considered, language, publication status) to be used as criteria for eligibility for the review |  |
|  |  |  |  |
| Information sources | 9 | Describe all intended information sources (such as electronic databases, contact with study authors, trial registers or other grey | Page 3 |
|  |  | literature sources) with planned dates of coverage |  |
|  |  |  |  |
| Search strategy | 10 | Present draft of search strategy to be used for at least one electronic database, including planned limits, such that it could be repeated | Page 3 |

Study records:

| Data | 11a | Describe the mechanism(s) that will be used to manage records and data throughout the review | Page 3 |
| --- | --- | --- | --- |
| management |  |  |  |
| Selection | 11b | State the process that will be used for selecting studies (such as two independent reviewers) through each phase of the review (that | Page 3 |
| process |  | is, screening, eligibility and inclusion in meta-analysis) |  |
| Data collection | 11c | Describe planned method of extracting data from reports (such as piloting forms, done independently, in duplicate), any processes | Page 4 |
| process |  | for obtaining and confirming data from investigators |  |
| Data items | 12 | List and define all variables for which data will be sought (such as PICO items, funding sources), any pre-planned data assumptions | Page 4 |
|  |  | and simplifications |  |
|  |  |  |  |
| Outcomes and | 13 | List and define all outcomes for which data will be sought, including prioritization of main and additional outcomes, with rationale | Page 3 |
| prioritization |  |  |  |
|  |  |  |  |
| Risk of bias in | 14 | Describe anticipated methods for assessing risk of bias of individual studies, including whether this will be done at the outcome or | Page 4 |
| individual studies |  | study level, or both; state how this information will be used in data synthesis |  |
|  |  |  |  |
| Data synthesis | 15a | Describe criteria under which study data will be quantitatively synthesised | Page 4 |
|  | 15b | If data are appropriate for quantitative synthesis, describe planned summary measures, methods of handling data and methods of | Page 4-5 |
|  |  | combining data from studies, including any planned exploration of consistency (such as I^2^, Kendall’s τ) |  |
|  | 15c | Describe any proposed additional analyses (such as sensitivity or subgroup analyses, meta-regression) | Page 5 |
|  | 15d | If quantitative synthesis is not appropriate, describe the type of summary planned | Page 4 |
|  |  |  |  |
| Meta-bias(es) | 16 | Specify any planned assessment of meta-bias(es) (such as publication bias across studies, selective reporting within studies) | Page 5 |
|  |  |  |  |
| Confidence in | 17 | Describe how the strength of the body of evidence will be assessed (such as GRADE) | Page 5 |
| cumulative evidence |  |  |  |

*** It is strongly recommended that this checklist be read in conjunction with the PRISMA-P Explanation and Elaboration (cite when available) for important clarification on the items. Amendments to a review protocol should be tracked and dated. The copyright for PRISMA-P (including checklist) is held by the PRISMA-P Group and is distributed under a Creative Commons Attribution Licence 4.0.**

*From: Shamseer L, Moher D, Clarke M, Ghersi D, Liberati A, Petticrew M, Shekelle P, Stewart L, PRISMA-P Group. Preferred reporting items for systematic review and meta-analysis protocols (PRISMA-P) 2015: elaboration and explanation. BMJ. 2015 Jan 2;349(jan02 1):g7647.*

Supplement 2 Search Strategy

| #1 | (((((((((Autografts[MeSH Terms]) OR (Autografts)) OR (autologous bone)) OR ((Allografts[MeSH Terms]) OR (Allografts))) OR ((Titanium[MeSH Terms]) OR (Titanium))) OR ((hydroxyapatite[MeSH Terms]) OR (hydroxyapatite))) OR ((Methylmethacrylate[MeSH Terms]) OR (Methylmethacrylate))) OR (((Ceramics[MeSH Terms]) OR (Ceramics)) OR (alumina ceramics))) OR (((polyetheretherketone [Supplementary Concept]) OR (polyetheretherketone)) OR (PEEK))) OR (synthetic grafts) |
| --- | --- |
| #2 | (((cranial defect) OR (skull defect)) OR (cranioplasty)) OR (cranial repair) |
| #3 | (((((((Clinical Trials, Randomized) OR (Trials, Randomized Clinical)) OR (Controlled Clinical Trials, Randomized)) OR (Randomized Controlled Trials[MeSH Terms])) OR ((Controlled Clinical Trial[MeSH Terms]) OR (Controlled Clinical Trial))) OR ((prospective study[MeSH Terms]) OR (prospective study))) OR ((double blind method[MeSH Terms]) OR (double blind method))) OR ((single blind method[MeSH Terms]) OR (single blind method)) |
| #4 | #1 AND #2 AND #3 |
